# Combining 3D printing and elastographic characterization: A novel sphenoid wing meningioma simulation model for neurosurgical training

**DOI:** 10.1007/s10143-025-04061-4

**Published:** 2026-01-15

**Authors:** Sifian Al-Hamid, Fränze Grellmann, Julius Reiser, Firat Taskaya, Vanessa M. Swiatek, Klaus-Peter Stein, Amir Amini, Karl Hartmann, Yuzhe Fan, Ali Rashidi, Claus-Dieter Ohl, I. Erol Sandalcioglu, Belal Neyazi

**Affiliations:** 1 Department of Neurosurgery, Otto-von-Guericke University, Leipziger Str. 44, Magdeburg, Saxony-Anhalt 39120 Germany; 2 Institute of Physics, Faculty of Natural Sciences, Otto-von-Guericke University, Universitätsplatz 2, 39106 Magdeburg, Germany

**Keywords:** Simulation, Surgery training, Meningioma, Shear-Wave-Elastography

## Abstract

High-fidelity simulation models are crucial for advancing neurosurgical training, particularly for complex skull base pathologies such as sphenoid wing meningiomas. This study introduces and validates a novel, cost-effective 3D-printed simulator specifically designed for sphenoid wing meningioma resection. A key innovation of this model is the integration of shear wave elastography (SWE) to enable objective biomechanical validation of tumor-mimicking materials. A patient-specific skull model was created using fused deposition modeling (FDM) 3D printing and paired with custom-molded tumor replicas. In a material validation substudy, 14 tumors made from seven candidate materials were assessed through SWE-based elasticity measurements and blinded evaluations by experienced neurosurgeons, focusing on tactile feedback, anatomical resemblance, and microsurgical handling. The most suitable material was used for subsequent training simulations involving final-year medical students, neurosurgical residents, and an expert surgeon. Surgical performance was objectively measured using the OSAMS scoring system, complemented by subjective participant surveys across three simulation rounds. SWE identified significant differences in elasticity among materials, enabling classification into soft, medium, and firm consistencies. A 10% cake glaze formulation demonstrated the highest surgical realism and mechanical similarity to real tumor tissue. Participants—especially novices—showed significant improvement in OSAMS scores across simulations, alongside a strong inverse correlation between OSAMS score and simulation time. Subjective evaluations confirmed the simulator’s high realism, educational value, and motivational impact. This study presents a validated, anatomically precise, and elastographically characterized simulator for sphenoid wing meningioma surgery. By combining affordable 3D printing with SWE-guided material selection, the model offers a reproducible platform for neurosurgical training with high educational and biomechanical fidelity. Its modular design and low cost make it well-suited for widespread academic implementation.

## Introduction

Meningiomas are the most common primary intracranial neoplasms, accounting for approximately 13–26% of all brain tumors [1]. These tumors originate from the arachnoid cap cells of the meninges and are typically benign, slow-growing, and well circumscribed. Despite their low histopathological grade, meningiomas often become clinically relevant due to their location and potential compression of critical neurovascular structures. Among them, sphenoid wing meningiomas pose a particular surgical challenge due to their proximity to the optic nerve, cranial nerves III–VI, and major cerebral arteries, including the internal carotid and middle cerebral arteries [2].

The consistency of meningiomas is a key factor influencing the resection strategy. Tumors may present as soft and suctionable or firm and fibrous, affecting the choice of surgical instruments and technique [3, 4]. While intraoperative assessment of tumor firmness has traditionally relied on the surgeon’s subjective impression, shear wave elastography (SWE) offers a non-invasive and objective method to quantify tissue stiffness [5]. SWE has been used to differentiate tumor consistency, assess brain-tumor interfaces, and guide surgical approaches in real time [5, 6].

Given the rarity and anatomical complexity of certain meningiomas—particularly those located at the skull base—there is an increasing need for realistic training tools. 3D-printed simulation models have emerged as a promising approach to bridge the gap between theoretical knowledge and surgical expertise [7, 8]. These models offer reproducible, patient-specific training environments without risk to the patient [9, 10]. However, only a limited number of existing simulators replicate the biomechanical properties of tumor tissue. Recent advances have begun to incorporate elastographic measurements to enhance simulation realism.

This study aims to develop and validate a high-fidelity 3D simulation model for sphenoid wing meningioma resection. By integrating shear wave elastography into the material selection process, the model not only replicates anatomical structures but also mimics the mechanical behavior of real tumor tissue. The study evaluates both the objective surgical performance of trainees and their subjective perceptions of realism and educational value.

## Materials and methods

### Model development

This study protocol follows the SQUIRE 2.0 guidelines. Approval for the use of anonymized patient data was granted by the local ethics committee at Otto-von-Guericke University Magdeburg (Ethics approval number: NeuroCAM 146/19). ChatGPT, a large language model developed by OpenAI, was used to assist with language editing and refinement of the manuscript. As no identifiable information was used in this publication, further consent for participation and publication was not required. All data were gathered with patient consent as part of routine clinical care.

The simulation model was designed to realistically replicate the anatomical and surgical characteristics of a sphenoid wing meningioma. The development process consisted of four key components: the skull, dura mater, surrounding brain tissue, and tumor.

#### Skull construction

**Fig. 1:**
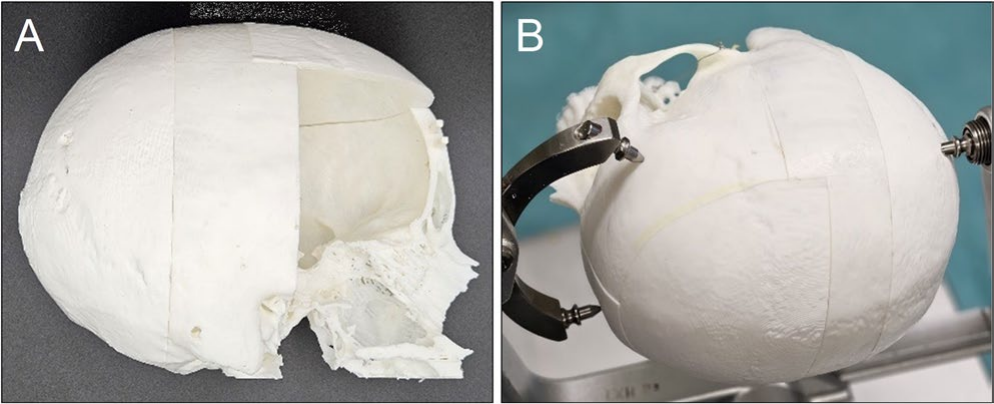
**A**. This is a representation of the five-part skull without the pterional section, allowing a view into the interior of the skull. **B**. The pinned skull with its five parts

Patient-specific cranial data was obtained from anonymized CT scans and segmented using InVesalius 3D software. The resulting files were refined in Meshmixer, a 3D-freeware by Autodesk and prepared for additive manufacturing with Bambu Studio. The skull was printed using Fused Deposition Modeling technology (FDM) with Polyactide-based (PLA-based) filament (PRIMA SELECT, PLA+, 1.75 mm) on a Bambu Lab P1S printer (Shenzhen, China 2020). For modular use, the skull was divided into five components, allowing the frontotemporal part—frequently altered during simulation—to be easily replaced after each trial **(**Fig. 1**)** [9, 10].

#### Dura mater and brain simulation

**Fig. 2:**
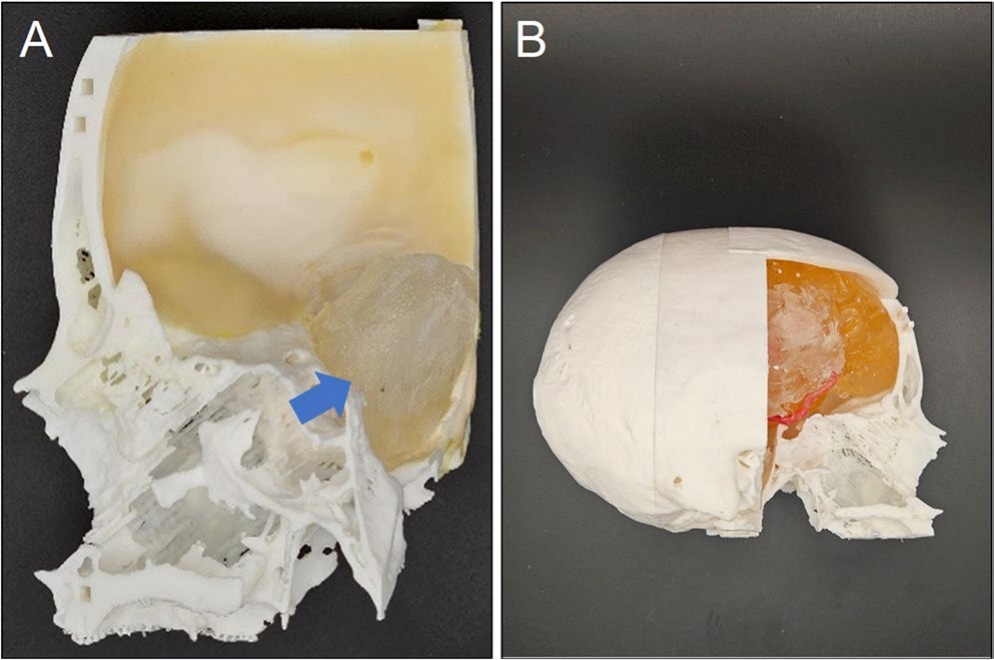
**A**. The pterional part of the skull from the inside, covered with latex as dura mater, and with a meningioma (marked by blue arrow). **B**. The brain with the Sylvian fissure and the arteries

The dura mater was represented using thickened Creative Latex (Lilatex LX30), which was poured into the inner surface of the skull and solidified using a latex catalyst **(**Fig. 2A**)**. Brain parenchyma, including the Sylvian fissure, was simulated using candle gel molded in custom silicone negatives. After cooling, the segments were assembled and affixed within the cranial cavity [9, 10]. The cerebral vessels, made from silicone, were sourced from a separate project partially published by Amini et al. [11] and further elaborated upon in the work of Reiser et al. [9] (Fig. 2B).

### Biomechanical material evaluation and tumor construction

A dedicated material validation study was conducted to assess the suitability of various tumor-mimicking substances through both subjective neurosurgical evaluation and objective SWE measurements.

#### Material evaluation

**Fig. 3:**
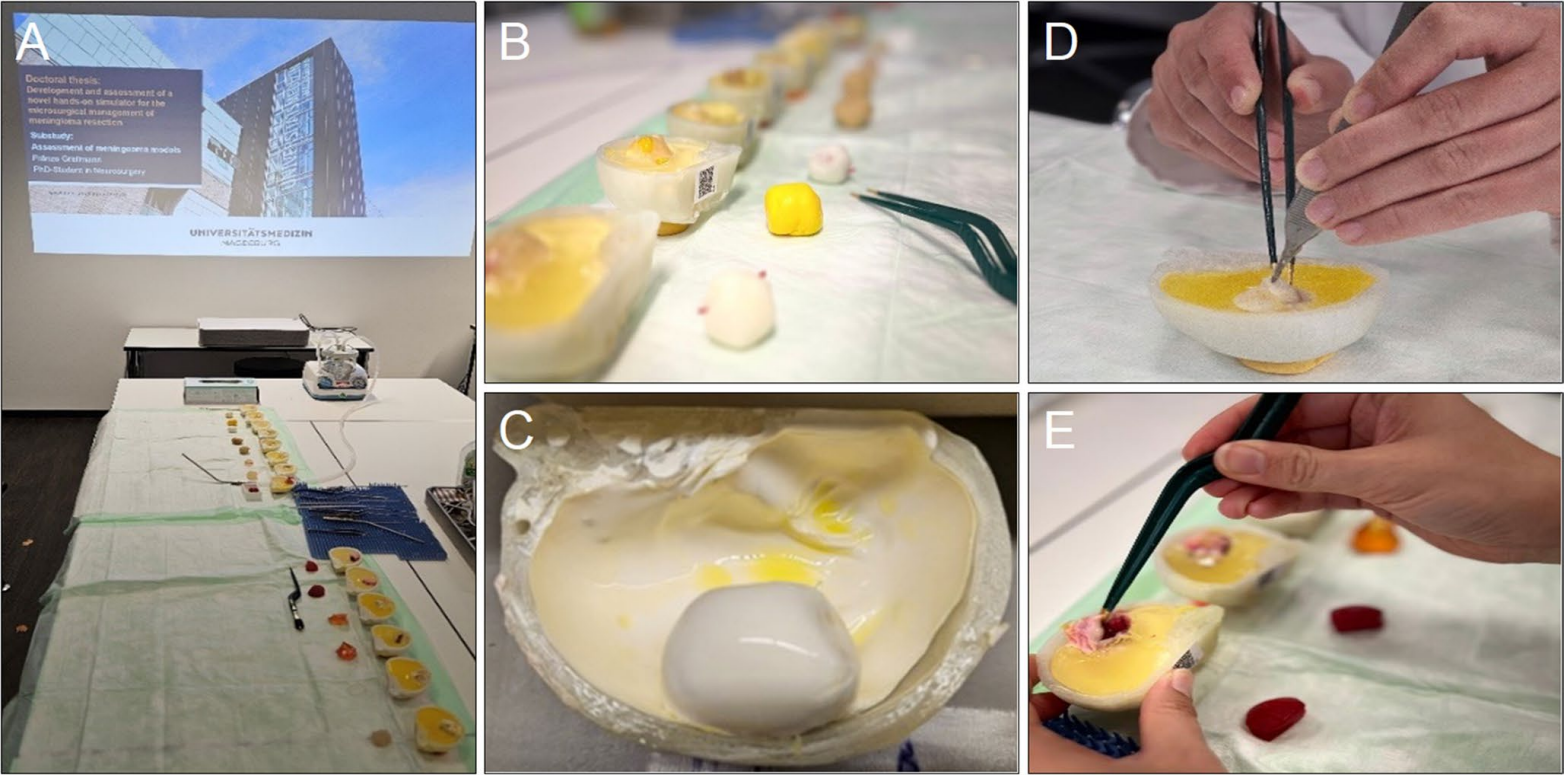
**A**. The setup for the subjective sub-study, including the explanatory PowerPoint, the instruments, the suction device, and the materials. **B**. The QR codes for the evaluation of the materials. **C**. The container shaped like a small skull, featuring the dura mater and the meningioma. **D**, **E**. The execution of the sub-studies by the experienced neurosurgeons

To identify the most suitable material for simulating meningioma tissue, a comparative substudy was conducted. Fourteen tumor models were fabricated using seven different materials (Table 1), each presented both embedded within a skull model and as an isolated sample (Fig. 3). Each tumor was sealed in a latex layer to simulate the tumor capsule and surrounded by candle gel to mimic brain parenchyma (Fig. 3A, B, C) [10]. Nine neurosurgeons, including department heads and senior consultants, evaluated the models based on tactile feedback, visual resemblance, and microsurgical manageability using a 10-point Likert scale (Fig. 3D, E).

**Table 1:** Overview of tested materials with terms as they were referred to in the manuscript, brand names/names of sources, and hydrogel concentrations

Materials	Brand name/Source	Concentrations of the hydrogels (in %)		
Goldbears	Haribo	50%	75%	
Polyvinylalcohol BF-17 (PVA)	COLLTEC PVA BF-17	10%	15%	
Cake glaze	Dr. Oetker	10%	20%	
Decorative cherrys	Gut&Günstig	100%		
Agar Agar	RUF	5%	10%	
Marzipan	Gut&Günstig	Pure (100%)	Mixed with pouder sugar (1:1)	
Fondant	RUF	fresh	stored 7 months	stored 1 month

**Fig. 4:**
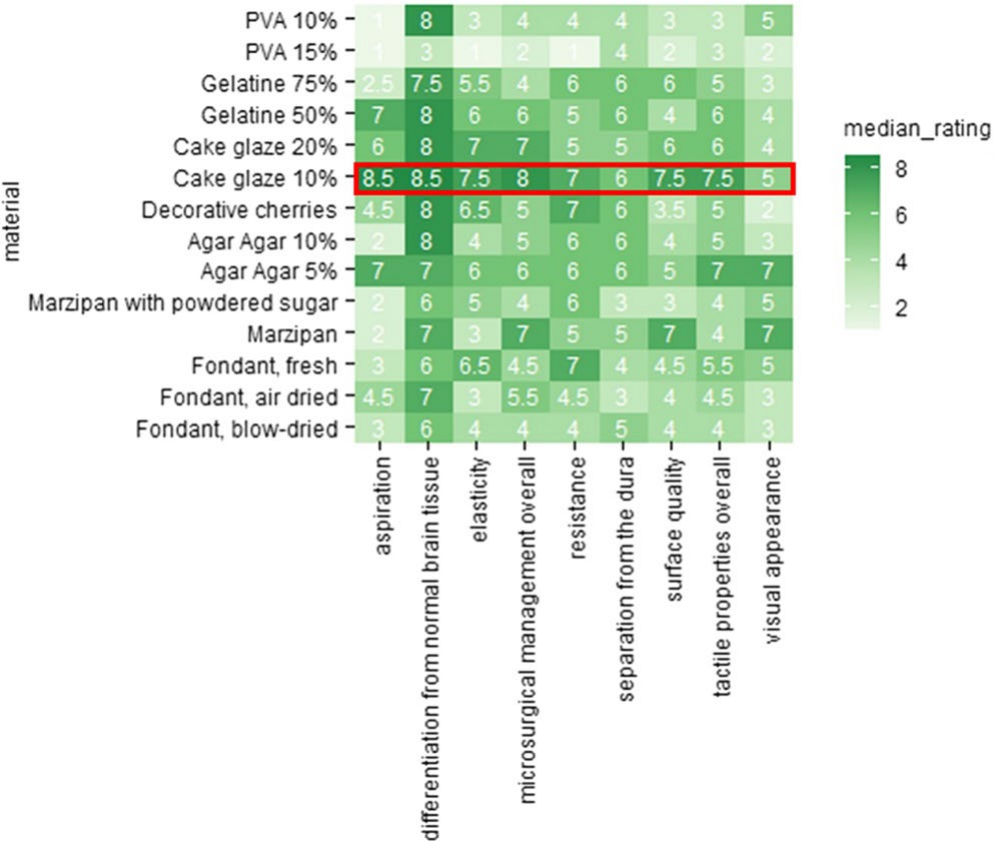
A. The subjective assessment of tactile properties: surface quality, resistance, and elasticity and the subjective assessment of microsurgical management: aspiration of the specimen, differentiation of the specimen from brain tissue, separation the specimen from the dura. Marked in red is the material that performed best: Cake Glaze 10%

Among all materials tested, 10% cake glaze achieved the highest overall rating, particularly for tactile realism. It outperformed 20% cake glaze and significantly surpassed materials like 15% Polyvinyl Alcohol (PVA) and air-dried fondant, which were rated lowest due to poor elasticity and resistance (Fig. 4).

10% cake glaze received the highest average score for surgical usability, including differentiation from brain tissue, suction compatibility, and separation from the dura. It slightly outperformed 20% cake glaze and 5% agar-agar (Fig. 4). Based on its superior tactile and surgical properties, 10% cake glaze was selected as the final meningioma simulation material.

As previously noted, firmness was not rated based on realism but rather on a scale from zero (very soft) to ten (very firm). Based on this, PVA and 10% agar-agar were classified as firm materials, while 5% agar and 10% cake glaze were considered soft. The remaining materials were categorized as medium-firm. Since real meningiomas vary in consistency, firmness ratings were excluded from material selection and served only for descriptive classification.

#### Biomechanical evaluation

SWE is a non-invasive ultrasound-based imaging technique used to measure the mechanical properties of soft tissues in real time. It quantifies tissue stiffness by analyzing the propagation speed of mechanically induced shear waves, providing an objective estimate of the Young’s modulus (E) in kilopascals (kPa) [12]. SWE has gained increasing importance in neurosurgery for assessing tumor consistency and guiding intraoperative strategy, especially in meningioma surgery [5, 6, 13]. According to the general principle of SWE described by Gennisson et al. [14], the Young’s modulus E can be estimated using the equation:

$$\:E={3pc}_{s}^{2}$$

where ρ is the tissue density and c_s_​ is the shear wave speed, and the Poisson’s ratio is assumed to be 0.5 for simplification. This relationship allows for real-time, quantitative characterization of tissue stiffness using ultrasound. Assuming a soft tissue density of approximately 1000 kg/m³, this formula enables real-time estimation of tissue stiffness from the measured wave propagation velocity.

In this study, SWE was used to evaluate the elasticity of five tumor-mimicking materials considered suitable for ultrasound-based simulation models. The SWE was performed using the Vantage 64LE system by Verasonics (Kirkland, USA). Here, materials that failed to generate measurable shear wave signals due to the lack of echogenicity or exhibited liquid-like behavior (e.g., 20% gelatin gel) were excluded. For the measurements, the acoustic radiation force push beam was focused at a distance of 20 mm from the transducer using 88 of the 128 active elements of the L7-4 ultrasound probe. The push duration was 200 µs and the push frequency was 5 MHz. A plane wave imaging method using single waves was used to track the shear waves, where the high frame rate of 40 kHz allows to cover a large range of shear speeds. Shear-wave elastography was performed at 25 °C. A fixed 300 mm² ROI was positioned centrally within each material, avoiding edges and air inclusions.

**Fig. 5:**
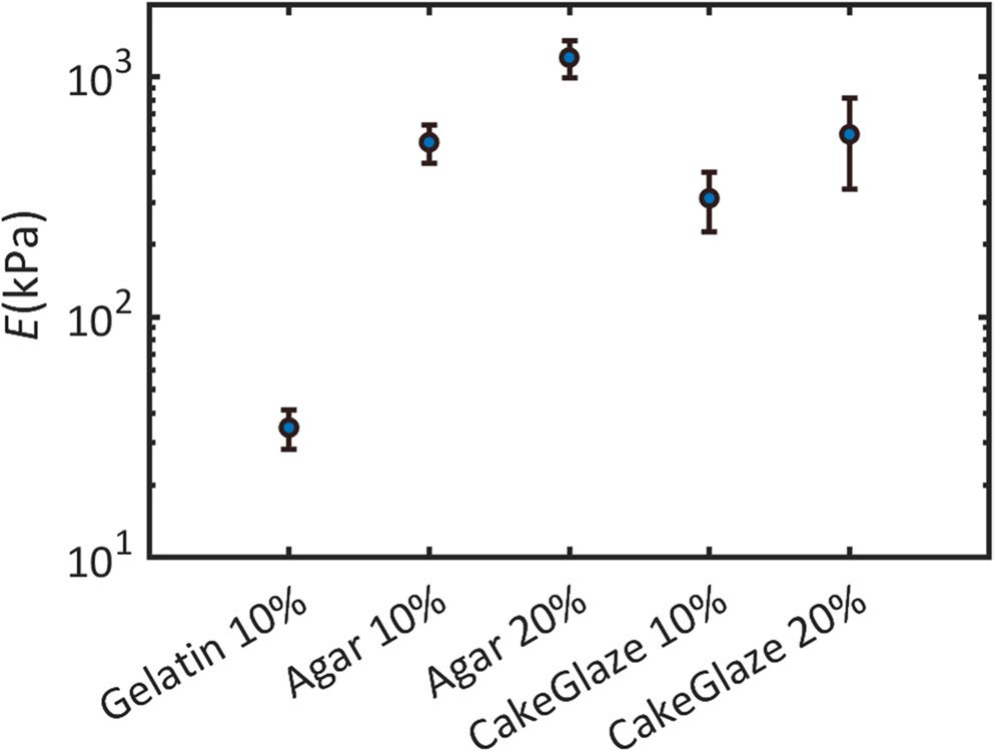
The Diagram shows the young’s modulus (E) values obtained for the five testable materials using the Vantage 64LE system by Verasonics

Figure 5 shows the young’s modulus (E) values obtained for the five testable materials. The results span over two orders of magnitude (approximately 20 kPa to over 1000 kPa), demonstrating substantial variability in elasticity:

- Gelatin 10% showed the lowest stiffness (~ 50 kPa), classifying it as a soft material.
- Agar 10%, Cake Glaze 10% and cake Glaze 20% displayed intermediate stiffness values (~ 100–500 kPa), placing them in a medium-firm category.
- Agar 20% exhibited the highest elasticity (> 1000 kPa), corresponding to very firm consistency.

The Young’s modulus values we measured for gelatin (≈ 1–100 kPa) fall within the ranges reported in the literature, including our prior data, supporting consistency [15–18]. By contrast, our agar formulation differs from commonly cited concentrations (< 4% w/w), and “CakeGlaze” is not a standardized material; therefore, direct benchmarking of these two materials against published datasets is not straightforward and adds limited interpretive value.

#### Tumor construction

The tumor model was based on MRI data of a patient diagnosed with a sphenoid wing meningioma. The tumor was manually segmented using medical imaging software to ensure accurate anatomical representation. Following segmentation, a 3D mesh of the tumor was generated and printed using PLA filament via FDM 3D printing technology.

To create a flexible, reusable version of the tumor, a negative silicone mold was produced from the printed 3D model. This mold served as a cast for filling various tumor-simulating materials. Once cured, the tumor replica was carefully inserted into the cranial cavity of the simulator and fixed in place using liquid latex, allowing for secure integration within the anatomical environment.

### Study design

The simulation process was designed to assess both the objective and subjective effectiveness of a 3D-printed sphenoid wing meningioma model in neurosurgical training. A total of seven participants were included: four final-year medical students without prior surgical experience, two neurosurgery residents, and one senior neurosurgeon with extensive surgical expertise.

Throughout the procedure, participants used standard neurosurgical instruments including bipolar forceps, suction, and microscissors. To ensure consistency, each session was timed and video-recorded. The resected skull segment was then replaced with a new one, enabling the reuse of the remaining skull base for subsequent simulations. All participants completed three consecutive simulations to assess performance progression and learning effects. The senior neurosurgeon completed only one procedure to serve as a reference.

**Fig. 6:**
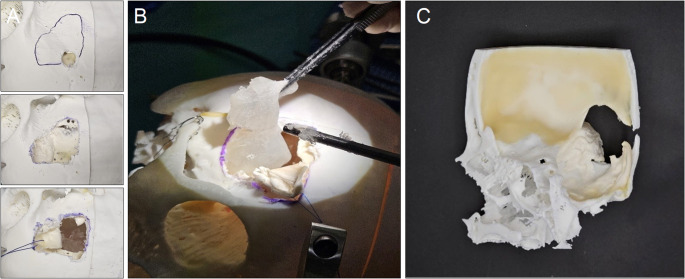
**A**. The pterional craniotomy with planning and dura opening. **B**. The resection of the meningioma either en bloc or via suction. **C**. The pterional skull segment after resection of the meningioma

Before the first simulation, all participants received a standardized briefing, including an introduction to the anatomy of sphenoid wing meningiomas, surgical objectives, and microscope handling. The simulation procedure involved performing a pterional craniotomy (Fig. 6A) and resecting the tumor model either en bloc or fragmented removal by suction, based on the participant’s preference (Fig. 6B).

**Fig. 7:**
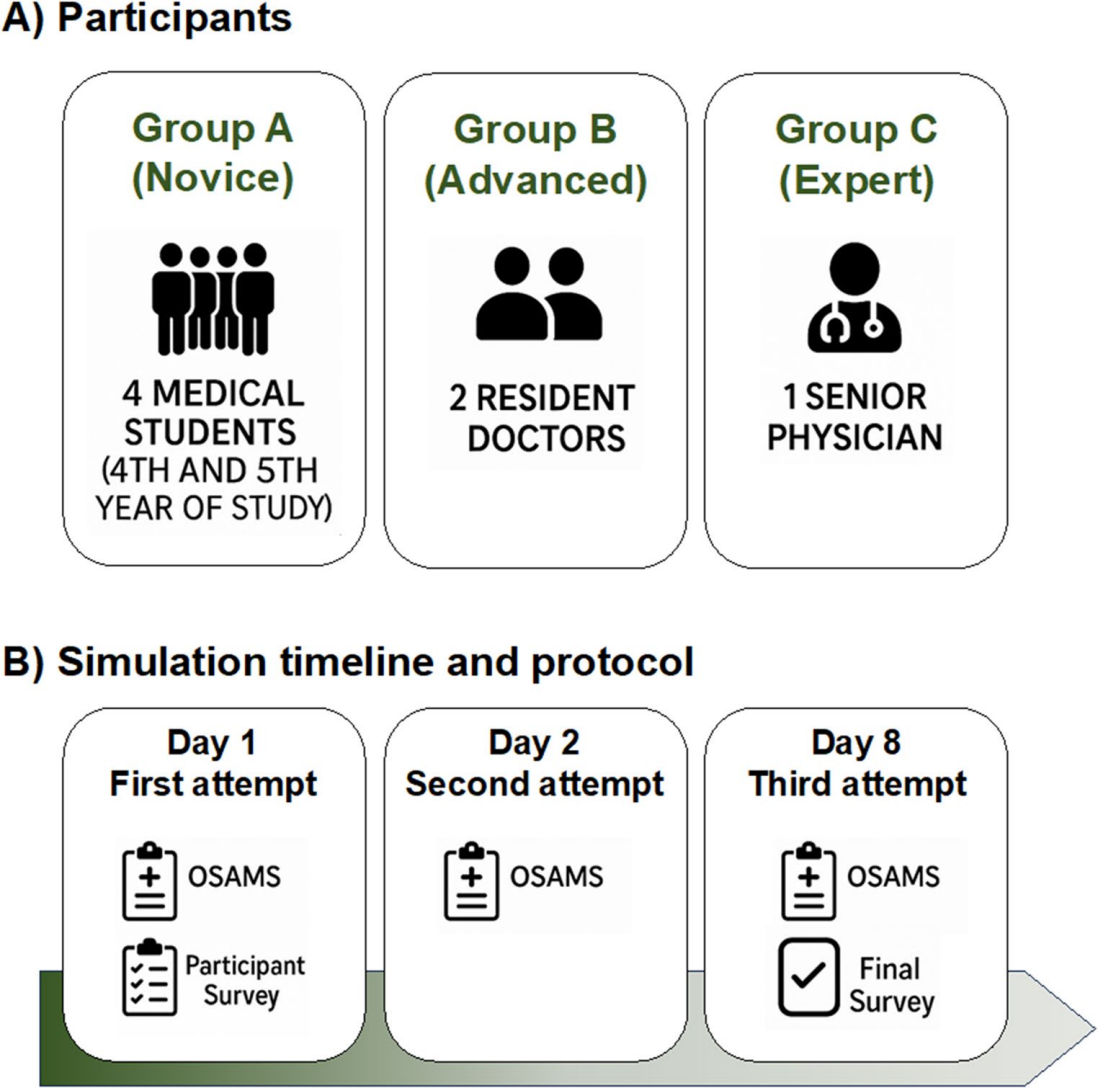
**A**. The maximum cumulative OSAMS scores achieved were by the medical students, the residents, and the experienced neurosurgeon, respectively. **B**. The progression of the three simulation attempts by the medical students, residents, and the experienced neurosurgeon was evaluated based on a key OSAMS criterion: time flow of operation and forward planning. **C**. The average times required by the medical students, residents, and the experienced neurosurgeon across all three simulation attempts were recorded

Each simulation was followed by an objective assessment using the Objective Structured Assessment of Meningioma Surgery (OSAMS). The OSAMS used in this study is an adaptation of the published Objective Structured Assessment of Aneurysm Clipping Skills (OSAACS) [9]. We retained the original 5-point anchored rating scale (1 = poor/unsafe, 3 = competent but improvable, 5 = optimal) and eight of the nine OSAACS domains (operator positioning and posture; use of the surgical microscope; knowledge of instruments; instrument handling; time and motion; time flow of operation and forward planning; respect for tissue; quality of dissection). The task-specific item “quality of aneurysm clipping” was modified to “quality of meningioma resection” to reflect the simulated procedure. Before scoring the participants, the attending neurosurgeon performed the procedure once and defined the behavioral anchors for score 5 in each item; this recording was used to train and calibrate the raters. All items were retained; given the small sample, item–total correlations are reported descriptively and were not used for item elimination (Fig. 7). Because the sample was small and nearly all students started from a comparable low baseline (floor effect), we did not fit a linear mixed-effects model. Instead, we analysed within-subject changes using Wilcoxon signed-rank tests for pairwise comparisons (Simulation 1 vs. 2, 2 vs. 3, 1 vs. 3) and a Friedman test for repeated measures across all three simulations. The level of significance was set at α = 0.05 (95% CI).

## Results

### Simulation performance

**Fig. 8:**
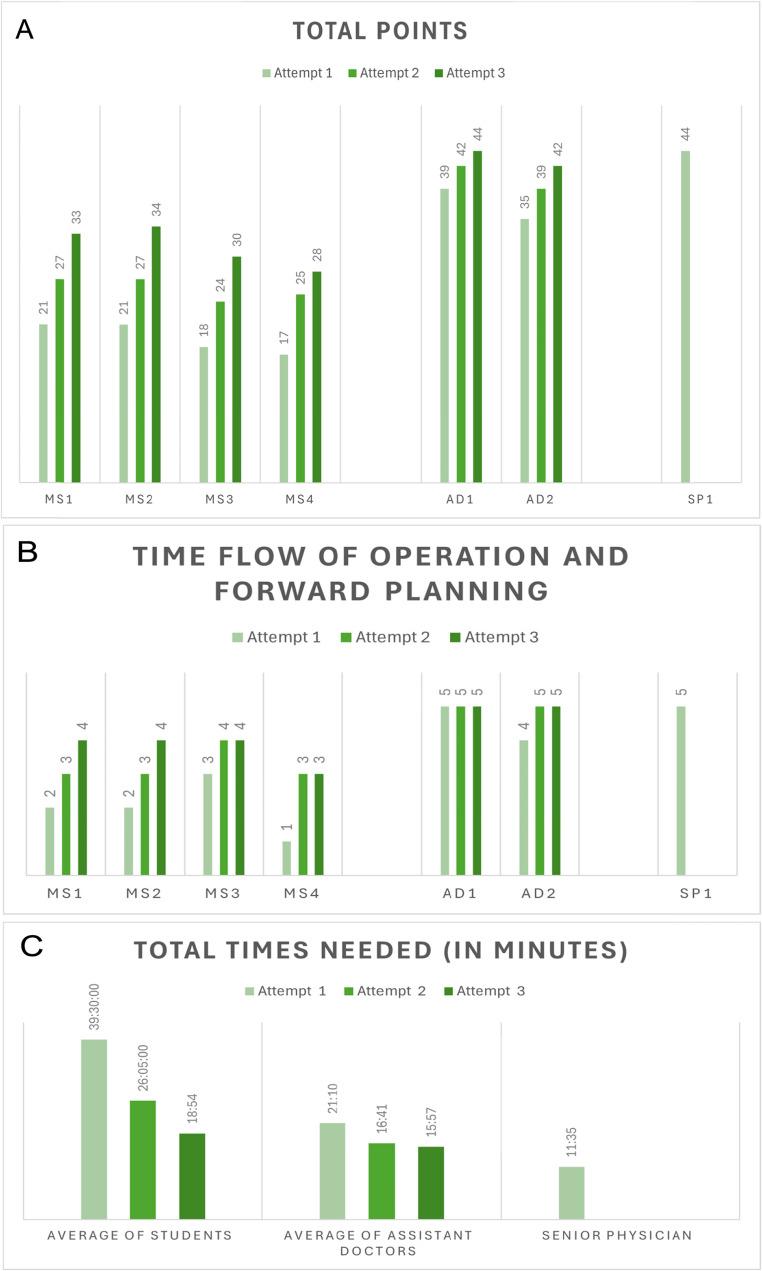
A. The maximum cumulative OSAMS scores achieved were by the medical students, the residents, and the experienced neurosurgeon, respectively. **B**. The progression of the three simulation attempts by the medical students, residents, and the experienced neurosurgeon was evaluated based on a key OSAMS criterion: time flow of operation and forward planning. **C**. The average times required by the medical students, residents, and the experienced neurosurgeon across all three simulation attempts were recorded

The four medical students, with no prior surgical experience, demonstrated the most substantial improvement over the three simulations. Their mean OSAMS scores increased from 19.25 (± 4.99) in the first trial to 31.25 (± 2.87) in the third, indicating significant skill acquisition over a short period (Fig. 8A, B).

The two neurosurgical residents, who already had prior surgical exposure, began at a higher baseline and also showed measurable improvement across simulations, though with a smaller absolute gain. The senior neurosurgeon, included for reference, scored 44 out of 45, confirming the upper performance limit of the model (Fig. 8A, B). In addition to improvements in surgical performance scores, participants required progressively less time to complete each simulation. This trend was observed consistently across all trainee groups during the three rounds (Fig. 8C).

### Subjective feedback

**Fig. 9:**
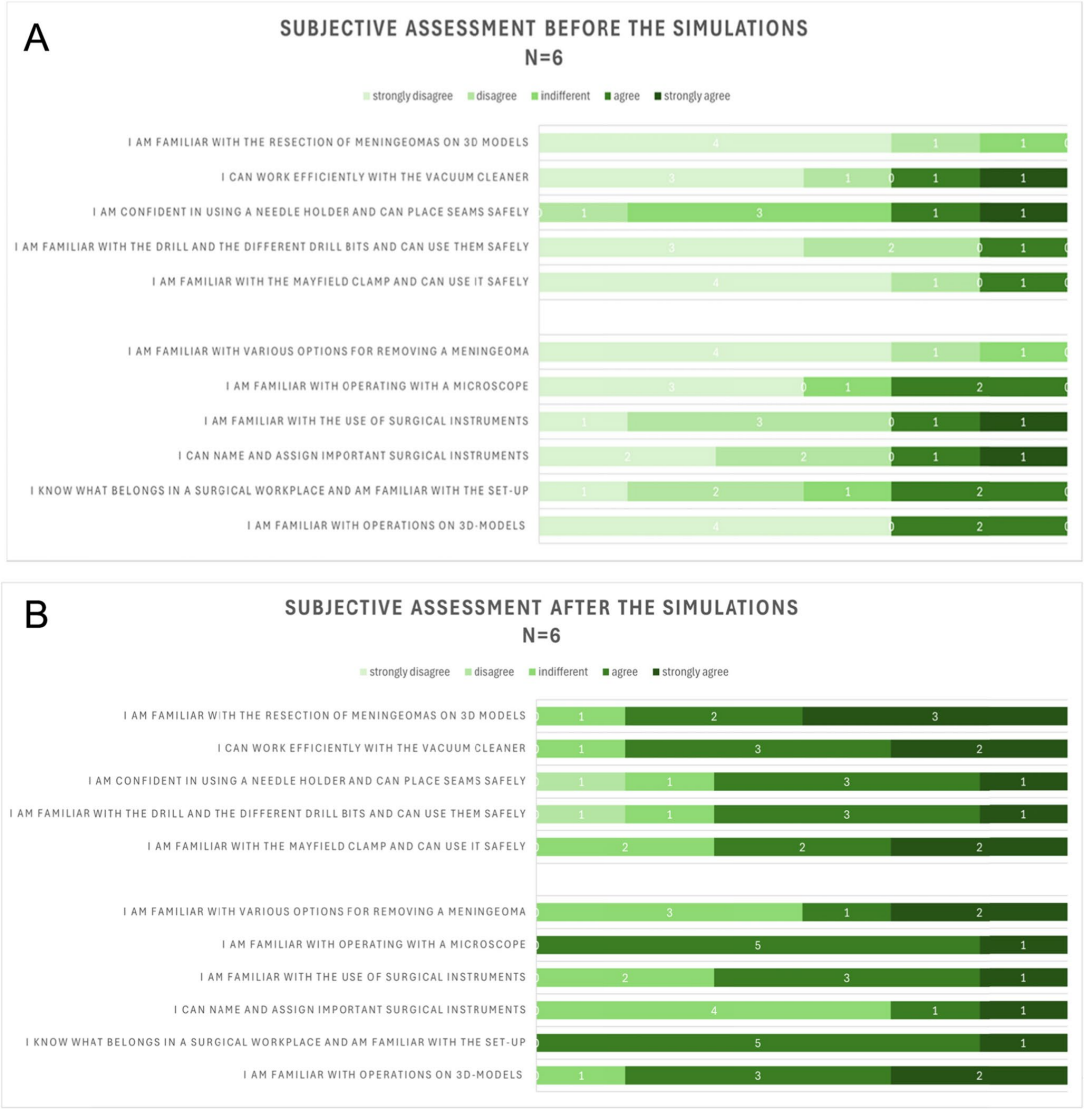
**A**. The subjective assessment was conducted prior to the first meningioma resection attempt to evaluate the participants’ initial confidence levels and expectations regarding the surgical procedure. **B**. The subjective assessment was repeated after the third meningioma resection attempt to evaluate the participants’ perceived improvements in surgical skills and their overall experience with the simulation model

Subjective feedback was collected through two standardized surveys. After the first simulation, the participant survey revealed that all participants perceived the model as highly realistic and valuable for training purposes. Statements regarding tactile feedback, surgical handling, and anatomical accuracy were consistently rated positively (Fig. 9A).

The final survey, administered after the third simulation, was designed as a pre-post comparison. Participants reported noticeable improvements in their surgical confidence and self-perceived competence. Medical students in particular noted that the model helped them overcome initial insecurities related to instrument handling and operative planning (Fig. 9B).

Moreover, participants emphasized the importance of having a realistic, pressure-free environment to practice and reflect on their performance. The model was unanimously described as beneficial not only for skill development but also for increasing motivation and interest in neurosurgery.

### Statistical analysis

Non-parametric analyses showed a significant improvement in performance across simulations (Friedman χ², *p* < 0.001). Wilcoxon signed-rank tests confirmed higher scores in Simulation 2 vs. 1, 3 vs. 2, and 3 vs. 1 (all *p* = 0.0312), with a median increase of 11.5 points from Simulation 1 to 3. Simulation time correlated strongly and negatively with total score (Spearman ρ = − 0.94, *p* = 0.0167), indicating that higher-performing participants completed the task faster.

### Cost analysis

The initial development of the simulation model amounted to approximately €139.30, primarily due to the use of high-quality silicone for creating reusable negative molds. Once these molds were available, the cost per simulation dropped significantly to around €3.00, as only a replaceable skull segment and a new tumor replica needed to be fabricated. Core equipment included a Bambu Lab P1S 3D printer (€799,00) and standard lab instruments. While the initial investment was moderate, the model’s durability and minimal recurring costs make it a highly economical training tool—particularly for institutions equipped with 3D printing infrastructure.

## Discussion

The findings of this study confirm the effectiveness of a cost-efficient, anatomically accurate 3D-printed simulator for neurosurgical training in the resection of sphenoid wing meningiomas. Participants, particularly medical students, showed a marked improvement in their OSAMS scores and self-confidence over the course of three simulations. This confirms the simulator’s capacity to enhance microsurgical skills in a structured and reproducible setting. The results are consistent with earlier work by Guo et al. and Al Ali et al., who demonstrated that 3D-printed simulators are particularly effective for early-stage trainees and enhance anatomical understanding and instrument handling [19, 20].

The model’s construct validity was supported by the high and consistent performance of the expert neurosurgeon, a method that has been used in previous validation studies such as those by Lan et al. [7]. Furthermore, a growing body of literature supports the educational value of simulation in neurosurgery, particularly when models are realistic and task-specific. Amini et al. reported that realistic vascular models significantly improved microsurgical dissection skills [7], while Rengier et al. highlighted the measurable improvements in performance and confidence across specialties when 3D-printed models were incorporated into surgical training [21]. The evaluation strategy, combining objective OSAMS scoring with subjective self-assessment, provided a comprehensive picture of training effectiveness and user perception. Such dual-layered evaluation has been advocated in recent simulation literature as a best practice for model validation [5, 22].

The positive effect of repetition and progressive exposure was also reflected in our findings. Durlak et al. emphasized that repeated practice is essential for the consolidation of procedural memory and surgical fluency [22]. In our study, repeated interaction with the same anatomical setup allowed participants to internalize both spatial relationships and motor tasks. The modular structure of the skull allowed for efficient replacement of the resection area, enabling repeated simulations with minimal material use. Compared to previously published models, the most prominent advantage of our simulator was the affordability: unlike simulators using multimaterial PolyJet technology, which can exceed 2000 USD per model as reported by Waran et al. [8], our model can be produced at a fraction of the cost, making it suitable for widespread use in academic settings. Given the limited availability of high-cost simulation technologies in low- and middle-income countries, the affordability of our model may offer a valuable alternative to support equitable access to surgical training.

A notable innovation in this study was the integration of SWE for the objective selection of tumor materials. While previous simulation studies have relied primarily on subjective evaluation, our method allowed for mechanical characterization of material stiffness and direct comparison to published SWE data for real meningiomas [4, 5, 23]. This added a level of biomechanical realism to the model that goes beyond purely visual or tactile resemblance. Additionally, the use of 10% gelatin-based cake glaze—selected through SWE validation—enabled realistic tumor resection either by en bloc removal or suction, thereby accommodating multiple surgical techniques and user preferences. In this study, SWE was used to characterize the mechanical properties of the training materials in an in-vitro setting, and the measurements should be interpreted as feasibility data rather than as definitive elasticity values. The approach currently lacks multiple replicates, dispersion metrics, and formal statistical comparison, and it is not yet anchored to in-vivo brain/tumor stiffness. Moreover, intraoperative shear wave elastography in cranial neurosurgery remains an adjunctive, off-label technique: it has been shown to be feasible through a craniotomy and to provide real-time stiffness maps in tumor and epilepsy surgery, but it is not yet a routine or guideline-based modality. Future work in our group will therefore pair intraoperative SWE with strain elastography in vivo to generate repeated measurements, quantify variability, and link material properties to clinically relevant elasticity ranges.

Nonetheless, this study has several limitations. The small sample size of seven participants reduces the statistical power of our conclusions. While clear trends were observed, larger-scale and potentially multicenter studies are needed to generalize the findings. Additionally, the absence of a control group prevents direct comparison with standard training approaches, which limits the ability to quantify the model’s added value over conventional methods.

Furthermore, the OSAMS assessments were performed by members of the study team, which introduces the risk of observer bias. The OSAMS instrument has not yet undergone formal validation; although it is closely modeled on the validated and previously used OSAACS [9], our scores still reflect expert raters’ subjective judgments. Accordingly, results should be interpreted cautiously, and future multi-center work will establish OSAMS validity (content, construct, criterion) and generalizability. Future work should consider blinded external evaluators to enhance objectivity. Another limitation is the short duration of the study. As in other simulator studies [13, 24], we were unable to determine whether the acquired skills were retained long-term or translated effectively into the operating room. Lastly, while subjective feedback was positive, it was predominantly provided by medical students. As their perspective lacks direct surgical experience, the assessment of realism may differ from that of more experienced neurosurgeons.

Future studies should aim to expand the participant pool and include control groups to allow for more robust comparisons. Our findings should be interpreted in light of the study’s design: a small, single-center educational cohort without a control or comparator modality, which limits external validity and precludes causal conclusions. Future work will require larger, multi-center samples and a randomized comparator (e.g., alternative training modalities) to confirm these effects and assess generalizability. Our cohort was heterogeneous (students, residents, one expert), which we used deliberately to assess known-groups validity of the simulation (students vs. residents). Future work will recruit a homogeneous resident sample, stratified by training year, to delineate learning curves and effect sizes more precisely. Longitudinal studies that assess skill retention weeks or months after simulation would also provide valuable insight into the lasting educational effect of such models. In terms of model design, further development could include integration of vascular and neural structures with distinct coloring and elasticity, as proposed by Zheng et al. [24], to further enhance anatomical realism and surgical applicability. Finally, scaling the model for use in endoscopic or minimally invasive approaches may broaden its relevance in neurosurgical training.

## Conclusion

This study presents a validated, low-cost 3D-printed simulation model for sphenoid wing meningioma resection, combining anatomical accuracy with objective material characterization through shear wave elastography. The model proved effective in improving microsurgical skills, particularly among novice trainees, and was rated highly for realism and educational value. Its modular design, affordability, and reproducibility make it a promising tool for neurosurgical training. Future studies should focus on long-term skill retention, integration into structured curricula, and broader clinical validation.

## Data Availability

No datasets were generated or analysed during the current study.

## Non-standard Abbreviations and Acronyms

FDM: Fused Deposition Modeling
SWE: Shear-Wave-Elastography
PLA: Polylactide
PVA: Polyvinyl Alcohol
OSAMS: Objective Structured Assessment of Meningioma Surgery
